# Employment of artificial intelligence for an unbiased evaluation regarding the recovery of right ventricular function after mitral valve transcatheter edge‐to‐edge repair

**DOI:** 10.1002/ejhf.3705

**Published:** 2025-06-09

**Authors:** Vera Fortmeier, Amelie Hesse, Teresa Trenkwalder, Márton Tokodi, Attila Kovács, Elena Rippen, Jule Tervooren, Michelle Fett, Gerhard Harmsen, Shinsuke Yuasa, Moritz Kühlein, Héctor Alfonso Alvarez Covarrubias, Moritz von Scheidt, Ferdinand Roski, Muhammed Gerçek, Tibor Schuster, N. Patrick Mayr, Erion Xhepa, Karl‐Ludwig Laugwitz, Michael Joner, Volker Rudolph, Mark Lachmann

**Affiliations:** ^1^ Department of General and Interventional Cardiology Heart and Diabetes Center Northrhine‐Westfalia, Ruhr University Bochum Bad Oeynhausen Germany; ^2^ Department of Internal Medicine I Klinikum rechts der Isar, TUM University Hospital, School of Medicine and Health, Technical University of Munich Munich Germany; ^3^ DZHK (German Center for Cardiovascular Research), partner site Munich Heart Alliance Munich Germany; ^4^ Department of Cardiovascular Diseases German Heart Center Munich, School of Medicine and Health, TUM University Hospital, Technical University of Munich Munich Germany; ^5^ Heart and Vascular Center Semmelweis University Budapest Hungary; ^6^ Department of Physics University of Johannesburg Auckland Park South Africa; ^7^ Department of Cardiovascular Medicine Okayama University Okayama Japan; ^8^ Department of Family Medicine McGill University Montreal Canada; ^9^ Institute of Anesthesiology German Heart Center Munich, School of Medicine and Health, TUM University Hospital, Technical University of Munich Munich Germany

**Keywords:** Echocardiography, Mitral regurgitation, Right ventricular dysfunction, Deep learning, Transcatheter edge‐to‐edge repair

## Abstract

**Aims:**

Long‐standing severe mitral regurgitation (MR) leads to left atrial (LA) enlargement, elevated pulmonary artery pressures, and ultimately right heart failure. While mitral valve transcatheter edge‐to‐edge repair (M‐TEER) alleviates left‐sided volume overload, its impact on right ventricular (RV) recovery is unclear. This study aims to use both conventional echocardiography and artificial intelligence to assess the recovery of RV function in patients undergoing M‐TEER for severe MR.

**Methods and results:**

The change in RV function from baseline to 3‐month follow‐up was analysed in a dual‐centre registry of patients undergoing M‐TEER for severe MR. RV function was conventionally assessed by measuring the tricuspid annular plane systolic excursion (TAPSE). Additionally, RV function was evaluated using a deep learning model that predicts RV ejection fraction (RVEF) based on two‐dimensional apical four‐chamber view echocardiographic videos. Among the 851 patients who underwent M‐TEER, the 1‐year survival rate was 86.8%. M‐TEER resulted in a significant reduction in both LA volume and estimated systolic pulmonary artery pressure (sPAP) levels (median LA volume: from 123 ml [interquartile range, IQR 92–169 ml] to 104 ml [IQR 78–142 ml], *p* < 0.001; median sPAP: from 46 mmHg [IQR 35–58 mmHg] to 41 mmHg [IQR 32–54 mmHg], *p* = 0.036). In contrast, TAPSE remained unchanged (median: from 17 mm [IQR 14–21 mm] to 18 mm [IQR 15–21 mm], *p* = 0.603). The deep learning model confirmed this finding, showing no significant change in predicted RVEF after M‐TEER (median: from 43.1% [IQR 39.1–47.4%] to 43.2% [IQR 39.2–47.2%], *p* = 0.475).

**Conclusions:**

While M‐TEER improves left‐sided haemodynamics, it does not lead to significant RV function recovery, as confirmed by both conventional echocardiography and artificial intelligence. This finding underscores the importance of treating patients before irreversible right heart damage occurs.

## Introduction

Patients with severe mitral regurgitation (MR) exhibit considerable heterogeneity in clinical presentation, influenced by the underlying aetiology, the extent of disease progression, and comorbidities. Similar to the staging classification for patients with severe aortic stenosis, which is based on the extent of extra‐aortic valve cardiac damage,^1^, ^2^ there has been a shift in perspective for patients with severe MR from a lesion‐centred approach to a more comprehensive view that considers multiple aspects of cardiopulmonary involvement.^3^, ^4^ Long‐standing severe MR leads to left atrial enlargement, elevated pulmonary artery pressure, and eventually, right heart failure. Notably, the development of extra‐mitral valve cardiac damage does not necessarily follow a linear progression from left heart dysfunction to pulmonary hypertension and ultimately right ventricular (RV) failure. Instead, this process can be aggravated by coexisting comorbidities. The multifactorial aetiology of RV dysfunction and the non‐linear progression of concomitant cardiac lesions are particularly common in elderly patients, who often present with multiple concurrent pathologies.

Historically, the significance of the right heart has been underestimated, largely due to early research that downplayed its role in overall cardiac function. Mid‐20th‐century studies, such as those by Starr *et al*.,^5^ demonstrated that even substantial damage to the RV wall in animal models resulted in only minor changes in venous pressure, leading to the belief that the right heart could sustain significant injury without severely affecting survival. This belief contributed to a long‐standing focus on the left heart, overshadowing the importance of the right heart. However, our understanding of the right heart's role in cardiovascular disease has evolved significantly. It is now recognized that RV function is a critical determinant of patient outcomes, particularly in conditions like severe MR.^6^, ^7^ Regardless of the underlying pathophysiology, assessing RV function is crucial for predicting survival in patients with severe MR.^8^, ^9^, ^10^ Yet, despite this recognition, diagnostic and therapeutic approaches for the right heart lag behind those for the left heart. For instance, tricuspid annular plane systolic excursion (TAPSE), a commonly used method for assessing RV function, has limitations in capturing the complex geometry and pathological remodelling of the right ventricle. Additionally, therapeutics effective for left heart failure, such as renin–angiotensin–aldosterone system inhibitors, do not necessarily translate to success in right heart failure, reflecting fundamental differences in the adaptive responses of the two ventricles.

Given the pivotal role of RV function in patient prognosis, accurate assessment is essential. While cardiac magnetic resonance (CMR) imaging is the gold standard for evaluating RV volumes and RV ejection fraction (RVEF),^11^ its use in routine clinical practice is limited by cost and accessibility. This gap presents an opportunity for artificial intelligence to enhance the utility of more widely available imaging modalities, such as conventional two‐dimensional (2D) echocardiography. Recent advancements, including the development of deep learning models for predicting RVEF from 2D echocardiographic videos, offer promising avenues for improving the assessment of right heart function in clinical settings.^12^ In a previous study, we demonstrated that deep learning‐derived RVEF outperformed several conventional metrics of RV function in predicting 1‐year mortality following mitral valve transcatheter edge‐to‐edge repair (M‐TEER).^13^

While M‐TEER aims to reduce the retrograde transmission of elevated left‐sided filling pressures, the potential for recovery of a pre‐damaged right heart remains poorly understood. Assessing cardiac recovery following M‐TEER is challenging, particularly because clinicians, inherently invested in their patients' well‐being, may unconsciously lean towards optimistic assessments. This optimism, while reflective of a hopeful therapeutic mindset, can inadvertently bias the assessment of a patient's actual clinical progress, and the unbiased assessment of a human operator is further challenged by the detectability of the edge‐to‐edge repair device on the echocardiogram. Therefore, this study aims to explore the use of artificial intelligence to provide an objective evaluation of RV function following M‐TEER. By applying a deep learning model to echocardiographic data, we seek to minimize subjective bias and ensure that clinical evaluations are rooted in consistent, data‐driven insights as shown in the assessment of MR severity itself.^14^, ^15^ This approach promises to deliver a more accurate assessment of RV function, ultimately enhancing patient care and providing clearer communication regarding the trajectory of cardiac function recovery after intervention.

## Methods

### Study population

This is a post‐hoc analysis of prospectively and systematically collected data from patients who underwent M‐TEER for symptomatic moderate‐to‐severe or severe MR at two high‐volume tertiary care centres in Germany between 2017 and 2023 (Heart and Diabetes Center Northrhine‐Westfalia and German Heart Center Munich). Baseline demographic and clinical characteristics were obtained from registry data or clinical records. All patients underwent transthoracic as well as transoesophageal echocardiography prior to M‐TEER. The number of devices implanted was determined by the discretion of the treating physician. Importantly, this dual‐centre registry is device‐agnostic, as it includes all patients who underwent M‐TEER at the two centres during the above‐mentioned period, irrespective of the device used. As an elderly patient population was studied, post‐procedural 1‐year all‐cause mortality was defined as a clinically meaningful primary outcome measure. Survival data were obtained from routine clinical follow‐ups, general practitioners, hospitals, and practice cardiologists or the German Civil Registry, meaning that no patient was lost to follow‐up. Moreover, clinical follow‐up, including transthoracic echocardiography, was routinely performed at 3 months after M‐TEER. The local ethics committees at each participating centre approved the collection and analysis of data in accordance with the Declaration of Helsinki, and all patients gave their written informed consent to be enrolled in an observational registry.

### Transthoracic echocardiography

All echocardiographic studies were performed by experienced institutional cardiologists during clinical routine using commercially available equipment. Echocardiographic measures were assessed according to the current guideline recommendations,^16^ and classification of MR severity was performed using an integrative, multiparametric four‐grade approach (mild ≙ I, moderate ≙ II, severe ≙ III, and massive ≙ IV). In addition, the aetiology of MR was defined as either primary (mitral valve prolapse +/− flail leaflet), secondary (ventricular dilatation, atrial dilatation, or both), or mixed. TAPSE was assessed in an apical four‐chamber echocardiographic view by placing the M‐mode cursor at the lateral tricuspid valve annulus and measuring the movement of the tricuspid annulus toward the apex during systole. Systolic pulmonary artery pressure (sPAP) was calculated by adding the peak transvalvular gradient across the tricuspid valve (estimated from the continuous‐wave Doppler profile of the tricuspid regurgitation jet) to the right atrial pressure. The latter was inferred from the diameter and collapsibility of the inferior vena cava, as outlined in contemporary guidelines.^16^, ^17^

### Transcatheter edge‐to‐edge repair

In all patients, the indication for M‐TEER was symptomatic moderate‐to‐severe or severe MR, and the local heart teams approved all procedures. Procedures were performed under general anesthesia with three‐dimensional (3D) transoesophageal echocardiographic and fluoroscopic guidance. Details of the procedures have been described previously.^18^

### Deep learning‐derived right ventricular ejection fraction assessment

Experienced echocardiographers reviewed all videos in each patient's echocardiographic studies and identified suitable 2D apical four‐chamber view recordings (either standard or RV‐focused, without color Doppler). Following the manual identification of suitable apical four‐chamber views, these videos were exported as de‐identified Digital Imaging and Communications in Medicine (DICOM) files and were processed using a previously published deep learning pipeline, coded in Python (Python Software Foundation, Wilmington, DE, USA). The architecture and design of the deep learning model for predicting RVEF have been previously described,^12^ and the deep learning pipeline, along with its requirements and instructions, is publicly available at https://github.com/rvenet/RVENet‐Demo. Briefly, the model comprises an ensemble of three spatiotemporal convolutional networks that were trained to predict 3D echocardiography‐derived RVEF from 2D apical four‐chamber view echocardiographic videos. Importantly, the model provided accurate predictions during both internal and external validation, identified RV dysfunction (i.e. an RVEF of <45%) with similar accuracy but higher sensitivity than expert human readers, and provided the same prognostic information as 3D echocardiography. Based on threshold definitions from 3D echocardiography guidelines, we defined preserved RV function as a predicted RVEF of ≥45%.^19^

### Statistical analysis

All statistical analyses were performed in R (R version 4.3.2; R Foundation for Statistical Computing, Vienna, Austria). Categorical data are presented as numbers and frequencies (%), while continuous data are expressed as median and interquartile range (IQR). Chi‐square or Fisher's exact tests were used to evaluate the association between categorical variables and independent‐samples Wilcoxon tests were used to compare continuous variables. Pairwise comparisons of pre‐ and post‐procedural data were performed using paired‐samples Wilcoxon test.

Univariable logistic regression analyses were conducted to identify clinical, laboratory, and echocardiographic predictors of reduction in pulmonary artery pressures following M‐TEER. Odds ratios and 95% confidence intervals (CIs) were calculated for each variable.

Survival was plotted using the Kaplan–Meier method, and the log‐rank test was applied to compare survival.

A *p*‐value of ≤0.05 was considered to indicate statistical significance.

## Results

### Clinical characteristics of the study population

A total of 851 patients who underwent M‐TEER for moderate‐severe and severe MR between 2017 and 2023 were included in this dual‐centre analysis. Baseline demographic, clinical, and echocardiographic data are summarized in *Tables* *1* and *2* (see also online supplementary *Tables Appendix* *S1* and *S2* for the complete list of devices used). The median age of the study population was 79.9 years (IQR 74.5–83.5 years), and 56.9% of patients were male (*Table* *1*). The majority of patients experienced severe exertional dyspnoea, with 71.3% in New York Heart Association (NYHA) functional class III and 11.5% in NYHA class IV. Additionally, patients presented with a median N‐terminal pro‐B‐type natriuretic peptide level of 2780 pg/ml (IQR 1280–6408 pg/ml). A reduction in MR severity to grade ≤II/IV was achieved in 792 of the 851 patients (93.1%), with a post‐procedural median mitral valve gradient of 3.0 mmHg (IQR 2.0–4.5 mmHg).

**Table 1:** Baseline demographics and pre‐procedural clinical characteristics

Patients, *n*	851
Age, years	79.9 (74.5–83.5)
Male sex, *n* (%)	484 (56.9)
BMI, kg/m^2^	25.0 (22.7–28.3)
Arterial hypertension, *n* (%)	706 (83.0)
Diabetes mellitus, *n* (%)	231 (27.1)
History of CAD, *n* (%)	523 (61.5)
History of COPD, *n* (%)	131 (15.4)
History of atrial fibrillation, *n* (%)	617 (72.5)
NYHA class, *n* (%)	
≤ II	146 (17.2)
III	607 (71.3)
IV	98 (11.5)
EuroSCORE II, %	5.13 (3.19–8.66)
eGFR, ml/min/1.73 m^2^	48 (36–63)
NT‐proBNP, pg/ml	2780 (1280‐6408)
Haemoglobin, g/dl	12.5 (11.0–13.7)
Aetiology, *n* (%)	
Primary	299 (35.1)
Secondary	457 (53.7)
Mixed	95 (11.2)

Categorical data are presented as *n* (%), while continuous data are expressed as median (interquartile range).

BMI, body mass index; CAD, coronary artery disease; COPD, chronic obstructive pulmonary disease; eGFR, estimated glomerular filtration rate; EuroSCORE II, European System for Cardiac Operative Risk Evaluation II; NT‐proBNP, N‐terminal pro‐B‐type natriuretic peptide; NYHA, New York Heart Association.

**Table 2:** Comparison of baseline and follow‐up echocardiographic characteristics

	Baseline (*n* = 851)	Follow‐up (*n* = 475)	*p*‐value
LVEF, %	50 (35–58)	49 (34–57)	0.002
LVEDD, mm	56 (50–63)	54 (48–62)	1.5 × 10^−8^
LVESD, mm	40 (34–50)	41 (33–51)	0.495
LVEDV, mL	140 (95–193)	125 (87–182)	0.082
LVEDV_indexed_, ml/m^2^	75.4 (52.8–104.1)	65.6 (47.7–94.4)	0.084
LVESV, ml	75 (42–127)	64 (40–105)	0.927
LVESV_indexed_, ml/m^2^	38.7 (23.4–67.3)	32.7 (21.8–57.3)	0.938
MV EROA, cm^2^	0.30 (0.21–0.42)		–
MR vena contract width, cm	0.7 (0.6–1.0)		–
MR volume, ml	48.2 (35–67)		–
MV gradient, mmHg		3 (2–4)	–
LA volume, ml	123 (92–169)	104 (78–142)	5.1 × 10^−5^
LA volume_indexed_, ml/m^2^	66.3 (51.1–89.5)	55.7 (42.0–74.8)	3.9 × 10^−5^
sPAP, mmHg	46 (35–58)	41 (32–54)	0.036
Right midventricular diameter, mm	31 (26–35)	31 (26–35)	0.096
Right midventricular diameter_indexed_, mm/m^2^	16.7 (14.5–19.2)	16.4 (13.8–18.9)	0.113
TAPSE, mm	17 (14–21)	18 (15–21)	0.603
Predicted RVEF, %	43.1 (39.1–47.4)	43.2 (39.2–47.2)	0.475
RA area, cm^2^	25 (19–31)	22 (17–30)	0.573
RA area_indexed_, cm^2^/m^2^	13.3 (10.5–16.6)	12.0 (9.2–15.6)	0.635
TAPSE/sPAP ratio, mm/mmHg	0.370 (0.274–0.517)	0.431 (0.310–0.591)	0.018
MR grade, *n* (%)			
II and II+/IV	39 (4.6)	440 (92.6)^a^	<2.2 × 10^−16^
III and III+/IV	492 (57.8)	32 (6.7)	<2.2 × 10^−16^
IV/IV	320 (37.6)	3 (0.6)	<2.2 × 10^−16^
TR grade ≥ III/IV, *n* (%)	191 (22.4)	76 (16.0)	8.0 × 10^−4^

Categorical data are presented as *n* (%), while continuous data are expressed as median (interquartile range). Indexed echocardiographic characteristics are normalized to body surface area.

EROA, effective regurgitant orifice area; LA, left atrial; LVEDD, left ventricular end‐diastolic diameter; LVEDV, left ventricular end‐diastolic volume; LVEF, left ventricular ejection fraction; LVESD, left ventricular end‐systolic diameter; LVESV, left ventricular end‐systolic volume; MR, mitral regurgitation; MV, mitral valve; RA, right atrial; RVEF, right ventricular ejection fraction; sPAP, systolic pulmonary artery pressure; TAPSE, tricuspid annular plane systolic excursion; TR, tricuspid regurgitation.

^a^ MR grade at follow‐up ≤I, II and II+/IV.

**Figure 1:**
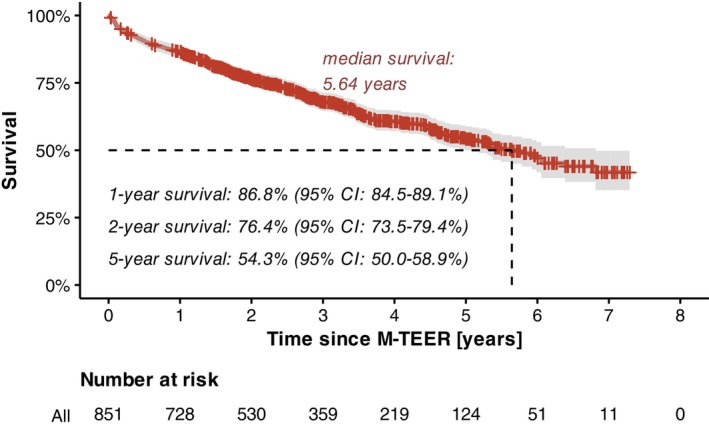
Survival plot for all patients enrolled in this dual‐centre registry of patients who underwent mitral valve transcatheter edge‐to‐edge repair (M‐TEER) between 2017 and 2023. CI, confidence interval.

**Figure 2:**
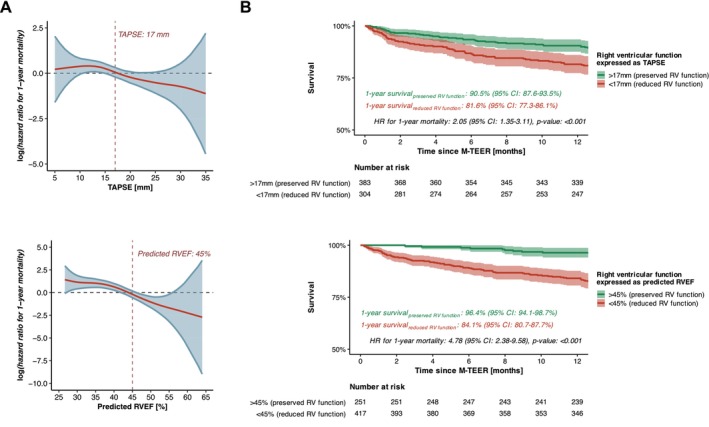
Survival of subgroups created based on right ventricular (RV) function assessed by conventional echocardiography using tricuspid annular plane systolic excursion (TAPSE) measurements or by deep learning‐derived prediction of right ventricular ejection fraction (RVEF) levels. (*A*) Spline plots illustrating the hazard ratio (HR) for 1‐year mortality after mitral valve transcatheter edge‐to‐edge repair (M‐TEER) as a function of TAPSE and predicted RVEF levels. (*B*) Kaplan–Meier curves of subgroups created based on RV function. CI, confidence interval.

During the study period, 310 deaths were documented among the enrolled patients. The remaining participants were followed for a median duration of 3.21 years (IQR 2.07–4.65 years). The overall 1‐year survival rate was 86.8% (95% CI 84.5–89.1%) (*Figure* *1*). Notably, stratification based on RV function, assessed using either conventional TAPSE measurements or deep learning‐enabled RVEF prediction, effectively identified high‐risk and low‐risk groups for 1‐year mortality following M‐TEER. Patients with reduced TAPSE (<17 mm) had a significantly higher risk of 1‐year mortality compared to those with preserved TAPSE (hazard ratio [HR] 2.05 [95% CI 1.35–3.11], *p* < 0.001), whereas the difference in mortality risk was even more pronounced between patients with reduced and preserved RVEF (HR 4.78 [95% CI 2.28–9.58], *p* < 0.001) (*Figure* *2*).

Follow‐up echocardiography was available for 475 patients (55.8%), typically performed at a median of 100 days (IQR 86–138 days) after the M‐TEER procedure. Importantly, patients who did not attend follow‐up echocardiography, including those with missing or unrecorded data, had a significantly higher risk of 1‐year mortality compared to those who did attend (HR 5.04 [95% CI 3.23–7.86], *p* < 0.001) (online supplementary *Figure Appendix* *S1*).

In this all‐comers registry, MR was classified as primary in 299 patients (35.1%), secondary in 457 patients (53.7%), and mixed in 95 patients (11.2%). As expected, patients with secondary MR exhibited more advanced left ventricular dysfunction, with significantly lower left ventricular ejection fraction (42% [IQR 30–53%] in secondary MR vs. 55% [IQR 50–60%] in primary MR; *p* < 2 × 10^−16^), and more severe left ventricular dilatation (left ventricular end‐diastolic volume: 162 ml [IQR 108–211 ml] vs. 118 ml [IQR 83–160 m]; *p* = 2.2 × 10^−6^) (online supplementary *Table* *S3* and *Figure* *S2*). Importantly, RV dysfunction was also more pronounced in secondary MR. This was reflected by both structural and functional parameters, including increased right midventricular diameter (32 mm [IQR 27–36 mm] vs. 29 mm [IQR 26–34 mm]; *p* = 0.003), lower TAPSE (17 mm [IQR: 13–20 mm] vs. 19 mm [IQR 15–22 mm]; *p* = 4.6 × 10^−7^), and significantly reduced deep learning‐predicted RVEF (42.0% [IQR 37.6–45.7%] vs. 45.1% [IQR 41.2–49.6%]; *p* = 3.8 × 10^−10^).

### Haemodynamic improvements after mitral valve transcatheter edge‐to‐edge repair

**Figure 3:**
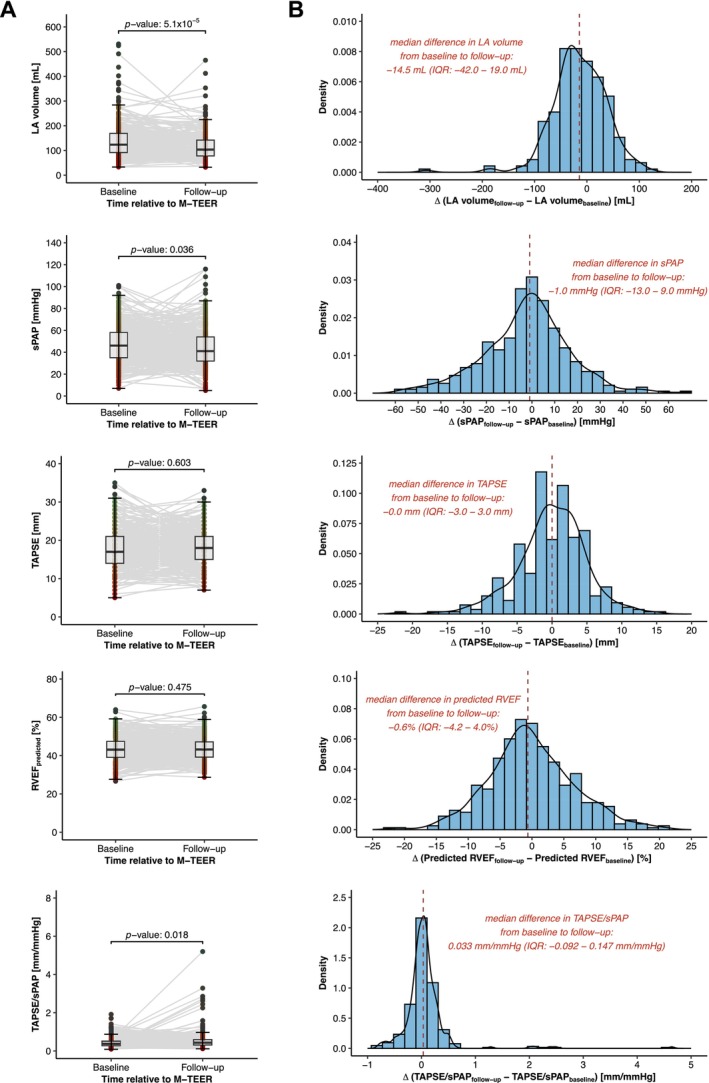
Structural and functional haemodynamic changes following mitral valve transcatheter edge‐to‐edge repair (M‐TEER). (*A*) Box plots with paired comparisons showing the changes in haemodynamic parameters from baseline to follow‐up after M‐TEER. (*B*) Density plots illustrating the distribution shift in haemodynamic parameters following M‐TEER. IQR, interquartile range; LA, left atrial; RVEF, right ventricular ejection fraction; sPAP, systolic pulmonary artery pressure; TAPSE, tricuspid annular plane systolic excursion.

**Figure 4:**
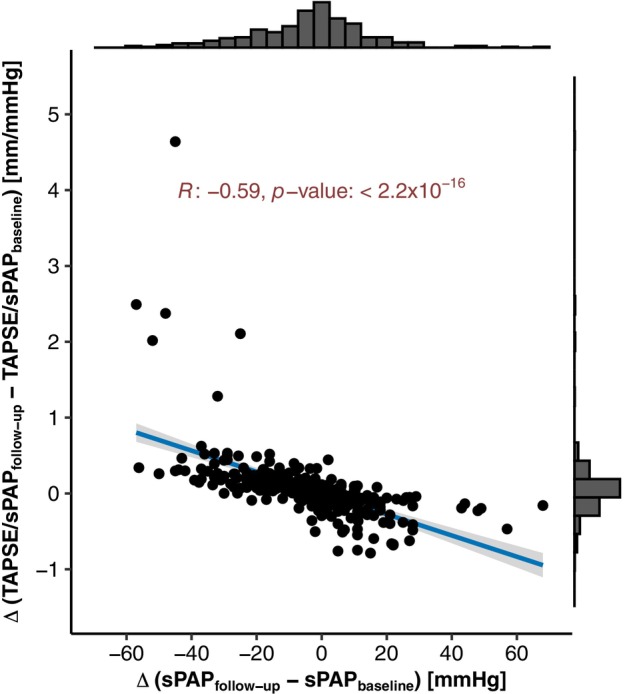
Correlation plot to analyse the association between the reduction of pulmonary artery pressure levels and the increase in right ventricular to pulmonary artery coupling following mitral valve transcatheter edge‐to‐edge repair. The blue line indicates the linear regression line, whereas the grey area denotes the 95% confidence interval. sPAP, systolic pulmonary artery pressure; TAPSE, tricuspid annular plane systolic excursion.

The most statistically significant change following M‐TEER was observed in left atrial volumes, which decreased from a median of 123 ml (IQR 92–169 ml) at baseline to 104 ml (IQR 78–142 ml) at follow‐up (*p* = 5.1 × 10^−5^) (*Table* *2* and *Figure* *3*). This reduction in left atrial volumes (further confirmed in an analysis of indexed echocardiographic parameters normalized to body surface area) (*Table* *2*), along with the improvement in retrograde transmission of filling pressures, was accompanied by a decrease in sPAP levels from 46 mmHg (IQR 35–58 mmHg) at baseline to 41 mmHg (IQR 32–54 mmHg) at follow‐up (*p* = 0.036). Importantly, RV function remained unchanged, as assessed by both conventional TAPSE (17 mm [IQR 14–21 mm] at baseline to 18 mm [IQR 15–21 mm] at follow‐up; *p* = 0.603) and deep learning‐derived RVEF prediction (43.1% [IQR 39.1–47.4%] at baseline to 43.2% [IQR 39.2–47.2%] at follow‐up; *p* = 0.475). Therefore, the observed improvement in RV to pulmonary artery (RV‐PA) coupling, expressed as an increase in the TAPSE/sPAP ratio from 0.370 mm/mmHg (IQR 0.274–0.517 mm/mmHg) at baseline to 0.431 mm/mmHg (IQR 0.310–0.591 mm/mmHg) at follow‐up (*p* = 0.018), was primarily driven by a reduction in pulmonary artery pressure levels following M‐TEER, as demonstrated by correlation analysis (Pearson's R: −0.59; *p* < 2.2 × 10^−16^) (*Figure* *4*).

Confirming the haemodynamic unloading of the right ventricle by amelioration of pulmonary artery pressure levels following M‐TEER, we also observed a significant reduction in the prevalence of severe tricuspid regurgitation (tricuspid regurgitation grade ≥ III/IV) following M‐TEER (from 22.4% at baseline to 16.0% at follow‐up; *p* = 8.0 × 10^−4^) (*Table* *2*). Despite unchanged intrinsic RV contractility and absence of structural reverse remodelling of the right ventricle (change in right midventricular diameter from baseline to follow‐up: 31 mm [IQR 26–35 mm] vs. 31 mm [IQR 26–35 mm]; *p =* 0.096), the improvement in tricuspid regurgitation severity is clinically relevant, as it enhances RV efficiency by reducing retrograde volume overload and increasing forward flow into the pulmonary circulation. This suggests that while RV contractile performance may not recover after M‐TEER, reductions in tricuspid regurgitation and pulmonary pressures can meaningfully improve RV haemodynamics and functional output.

Notably, RV function (TAPSE and deep learning‐predicted RVEF) correlated significantly with left heart function (expressed as left ventricular ejection fraction; *p* <2.2 × 10^−16^) but not with sPAP levels (*p* = 0.373 and 0.516, respectively) (online supplementary *Figure* *S3A*). Consequently, no significant differences in RV function were observed across pulmonary hypertension severity groups. Moreover, no RV function improvement (TAPSE or predicted RVEF) was detected in patients with mild, moderate, or severe pulmonary hypertension following M‐TEER (online supplementary *Figure* *S3B*).

Importantly, in patients with secondary MR, neither left ventricular function (as measured by left ventricular ejection fraction) nor RV function (as assessed by TAPSE and deep learning‐predicted RVEF) improved significantly following M‐TEER. Instead, these patients exhibited persistent biventricular dysfunction at follow‐up. At the same time, patients with primary MR showed a statistically significant but clinically negligible decline in predicted RVEF, from 45.1% (IQR 41.2–49.6%) at baseline to 44.2% (IQR 41.1–48.8%) at follow‐up (*p* = 0.022), while TAPSE remained unchanged (19 mm [IQR 15–22 mm] vs. 19 mm [IQR:17–23 mm]; *p* = 0.618) (online supplementary *Figure* *S2*).

As further tested by regression analysis, baseline MR severity was not significantly associated with changes in either TAPSE (*p* = 0.387) or deep learning‐predicted RVEF (*p* = 0.090) following M‐TEER.

### Predictors of systolic pulmonary artery pressure reduction after mitral valve transcatheter edge‐to‐edge repair

Considering the importance of reducing sPAP levels to alleviate the workload on a potentially compromised right heart, we performed a univariable regression analysis to identify the predictors of sPAP reduction following M‐TEER. Among various clinical, laboratory, and echocardiographic parameters, only arterial hypertension and a history of atrial fibrillation emerged as statistically significant predictors (*Table* *3*).

**Table 3:** Univariable analysis of clinical, laboratory, and echocardiographic predictors for reduction of pulmonary artery pressure following mitral valve transcatheter edge‐to‐edge repair

	OR (95% CI)	*p*‐value
Age (per 1‐year increment)	0.99 (0.96–1.01)	0.382
Sex (female as reference)	0.99 (0.67–1.48)	0.966
BMI (per 1 kg/m^2^ increment)	1.0 (0.93–1.07)	0.971
Arterial hypertension	0.50 (0.27–0.90)	0.024
History of CAD	1.07 (0.72–1.60)	0.731
History of COPD	0.71 (0.40–1.26)	0.243
History of atrial fibrillation	0.59 (0.37–0.93)	0.024
NYHA class (per severity grade increment)	0.90 (0.63–1.28)	0.561
NT‐proBNP (per 1000 pg/ml increment)	1.03 (0.99–1.08)	0.101
LVEF (per 1% increment)	0.99 (0.98–1.01)	0.306
Preprocedural MR severity (per severity grade increment)	1.07 (0.74–1.54)	0.716
LA volume (per 1 ml increment)	1.0 (0.99–1.00)	0.355
Right midventricular diameter (per 1 mm increment)	0.99 (0.95–1.03)	0.565
TAPSE (per 1 mm increment)	1.03 (0.99–1.08)	0.135
Predicted RVEF (per 1% increment)	1.0 (0.96–1.03)	0.815
Pre‐procedural TR severity (per severity grade increment)	1.20 (0.95–1.53)	0.124
RA area (per 1 cm^2^ increment)	0.98 (0.96–1.00)	0.088
Post‐procedural MR severity (per severity grade increment)	0.89 (0.65–1.22)	0.471
Post‐procedural MV gradient (per 1 mmHg increment)	0.93 (0.82–1.05)	0.264

BMI, body mass index; CAD, coronary artery disease; CI, confidence interval; COPD, chronic obstructive pulmonary disease; LA, left atrial; LVEF, left ventricular ejection fraction; MR, mitral regurgitation; MV, mitral valve; NT‐proBNP, N‐terminal pro‐B‐type natriuretic peptide; NYHA, New York Heart Association; OR, odds ratio; RA, right atrial; RVEF, right ventricular ejection fraction; TAPSE, tricuspid annular plane systolic excursion; TR, tricuspid regurgitation.

**Figure 5:**
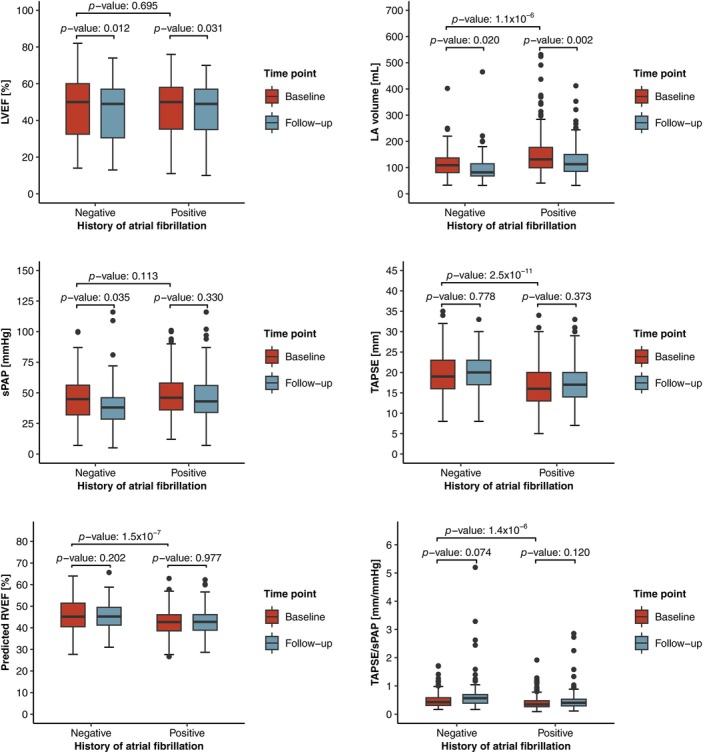
Paired box plots comparing structural and functional haemodynamic changes following mitral valve transcatheter edge‐to‐edge repair (M‐TEER) stratified by history of atrial fibrillation. LA, left atrial; LVEF, left ventricular ejection fraction; RVEF, right ventricular ejection fraction; sPAP, systolic pulmonary artery pressure; TAPSE, tricuspid annular plane systolic excursion.

At baseline, compared to patients without a history of atrial fibrillation, those with atrial fibrillation presented with significantly larger left atrial volumes (median: 132 ml [IQR 99–177 ml] vs. 109 ml [IQR 81–137 ml], *p* = 1.1 × 10^−6^), similar sPAP levels (median: 46 mmHg [IQR 36–58 mmHg] vs. 45 mmHg [IQR 32–56 mmHg], *p* = 0.113), and consistently poorer RV function, as measured by either TAPSE (median: 16 mm [IQR 13–20 mm] vs. 19 mm [IQR 16–23 mm], *p* = 2.5 × 10^−11^) or deep learning‐predicted RVEF (median: 42.7% [IQR 38.6–46.1%] vs. 45.2% [IQR 40.5–51.4%], *p* = 1.5 × 10^−7^) (*Figure* *5*). While left atrial volumes were reduced following M‐TEER in both patients with and without a history of atrial fibrillation, only those without atrial fibrillation experienced a reduction in sPAP levels (from a median of 45 mmHg [IQR 32–56 mmHg] at baseline to 38 mmHg [IQR 29–46 mmHg] at follow‐up, *p* = 0.036), confirming the findings from the univariable analysis. Importantly, in both subgroups (with and without atrial fibrillation), RV function, assessed conventionally by TAPSE or by deep learning‐predicted RVEF, showed no significant changes after M‐TEER.

## Discussion

### Novelty: first objective assessment of right ventricular function recovery after mitral valve transcatheter edge‐to‐edge repair using deep learning

This is the first study to use a deep learning model for the unbiased assessment of RV function recovery following M‐TEER. Our findings demonstrate that, while M‐TEER elicits positive effects on left‐sided haemodynamics – such as reductions in left atrial volume and pulmonary artery pressures – RV function remains unchanged from baseline to follow‐up. This finding was consistently observed using both conventional echocardiographic assessments and deep learning‐enhanced echocardiography, adding further credibility to our conclusion. Notably, the applied deep learning model is not influenced by subjective biases or ‘wishful thinking’, which may cause clinicians to overestimate RV function, especially in patients with clearly visible edge‐to‐edge repair devices. As such, deep learning‐enhanced echocardiography proves particularly useful in resolving controversial issues, such as the debated potential for RV functional recovery after M‐TEER.

### Can baseline differences in right ventricular function explain discrepant outcomes in the COAPT and MITRA‐FR trials?

Our findings underscore the growing recognition of RV function as a crucial determinant of outcomes in patients with valvular heart disease, particularly MR. Demonstrating that impaired RV function at baseline does not improve after M‐TEER highlights the need for careful consideration of RV function during patient selection for this procedure. This could help identify a potential threshold below which patients may no longer derive a clinically relevant benefit from M‐TEER. The understanding that RV function does not recover after M‐TEER, and thus baseline RV function determines prognosis, may reconcile the divergent findings from two randomized trials in patients with secondary MR undergoing M‐TEER: the COAPT and MITRA‐FR studies.^20^, ^21^ Notably, moderate to severe RV dysfunction was a key exclusion criterion in the COAPT trial, which may have contributed to its more favourable outcomes in terms of lower hospitalization rates for heart failure and reduced all‐cause mortality within 2 years compared to medical therapy alone. In contrast, the MITRA‐FR trial included patients with RV dysfunction and showed no significant difference between those treated with M‐TEER and those receiving medical therapy alone. Unfortunately, no secondary analysis or meta‐analysis has compared outcomes in relation to RV function between the COAPT and MITRA‐FR trials, and to our knowledge, data on RV function in these studies are not publicly available. An individual patient‐level meta‐analysis would be needed to gain deeper insights into the impact of RV function on outcomes in these cohorts. Additionally, knowing that patients with RV dysfunction were excluded from the COAPT trial and that RV systolic pressure was higher in MITRA‐FR than in COAPT (mean values ± standard deviation: 44.3 ± 14 vs. 54 ± 14 mmHg), suggesting a higher afterload burden on the right ventricle with subsequent RV damage in the MITRA‐FR cohort, may partially explain the discrepant outcomes between these trials. Consistent with our observations of irreversible RV structural and functional impairment, the study by Lurz *et al*.^22^ analysed the evolution of cardiac damage following M‐TEER using CMR, the gold standard for assessing RV structure and function. The authors reported that in 20 patients with either primary (*n* = 5 patients) or secondary MR (*n* = 15 patients), neither RV volumes nor CMR‐derived RVEF changed significantly after M‐TEER (mean RVEF ± standard deviation from baseline to follow‐up: 42 ± 9% vs. 43 ± 11%).

### Right ventricular function and pulmonary artery coupling: insights into haemodynamic changes following mitral valve transcatheter edge‐to‐edge repair

Aside from TAPSE, which is the most frequently used marker of RV function, more sophisticated yet conventional echocardiographic parameters exist for assessing RV function in patients with complex pathologies like MR. These include RV fractional area change and peak systolic velocity of the lateral tricuspid valve annulus. Even when comparing various RV function parameters in patients undergoing M‐TEER, van Riel *et al*.^23^ have demonstrated that RV function does not improve significantly at 1‐month follow‐up, whereas sPAP levels do decrease (mean values ± standard deviation: from 44 ± 13 mmHg at baseline to 40 ± 10 mmHg at 1‐month follow‐up; *p* < 0.01). The amelioration of pulmonary artery pressures, along with an absent recovery or even further decline of RV function, was also observed in a study on left ventricular and RV haemodynamic trajectories among patients receiving transcatheter mitral valve replacement.^24^ This emphasizes the importance of interpreting RV function in relation to its afterload, as indicated by sPAP levels. Consequently, RV‐PA coupling, commonly expressed as the TAPSE/sPAP ratio, has emerged as a prognostic marker in patients undergoing M‐TEER.^25^, ^26^ Interestingly, RV function, as measured by the TAPSE/sPAP ratio, can improve in up to 66% of patients following M‐TEER, and this improvement in TAPSE/sPAP at follow‐up was independently associated with reduced risk of mortality.^10^ Again, the recovery of RV‐PA coupling was primarily driven by an effective M‐TEER procedure (characterized by an optimal result in terms of residual MR and low post‐procedural transmitral gradients), which subsequently led to a reduction in pulmonary artery pressures and RV afterload. These findings suggest that pulmonary hypertension alone may not be the primary driver of RV dysfunction in this population. Instead, persistent RV dysfunction after M‐TEER likely reflects irreversible myocardial remodelling, underscoring the need for earlier intervention before significant right heart impairment occurs. This aligns with previous studies demonstrating that while sPAP reductions after M‐TEER alleviate RV afterload, they do not necessarily translate into RV functional recovery, emphasizing the importance of identifying patients before irreversible RV damage occurs. A similar mechanism, where an improvement in RV‐PA coupling is largely linked to a reduction in pulmonary artery pressures, has also been described in patients undergoing transcatheter aortic valve replacement.^27^ As such, the improved RV‐PA coupling may reflect a more favorable haemodynamic environment that allows the right ventricle to operate more efficiently, even in the absence of structural reverse remodelling. The concurrent reduction in severe tricuspid regurgitation supports this interpretation, as the weakened right ventricle can now eject more effectively into the pulmonary circulation rather than regurgitating into the right atrium. Although these changes may not constitute true ‘recovery’ of RV function in the classical sense, they represent improved RV efficiency and forward flow, which could have important clinical implications, particularly for symptom relief and long‐term prognosis.

### Potential implications for optimal timing of mitral valve transcatheter edge‐to‐edge repair: contextualizing lessons from RESHAPE‐HF2 in light of our findings

The RESHAPE‐HF2 trial recently demonstrated that even patients with moderate to severe functional MR may benefit from early M‐TEER, with significant reductions in heart failure hospitalizations and cardiovascular mortality compared to medical therapy alone.^28^ However, our findings suggest that the potential for RV recovery may not be primarily determined by MR severity at baseline. In our cohort, neither baseline MR severity nor MR aetiology showed a significant association with improvement in RV function after M‐TEER, as evidenced by both conventional TAPSE and deep learning‐predicted RVEF. These results imply that extra‐mitral valve cardiac damage, particularly irreversible RV remodelling, might be a more decisive factor for long‐term outcomes and functional recovery than MR severity *per se*. This supports the notion, as already shown by previous unsupervised machine learning‐based phenotyping studies,^8^ that timely identification of patients before the onset of significant RV involvement may be crucial. Accordingly, these data highlight the need for comprehensive, early risk stratification that incorporates not just MR severity but also right‐sided cardiac structure and function to guide optimal timing of intervention.

### Managing atrial fibrillation and hypertension to optimize right ventricular afterload and haemodynamic outcomes after mitral valve transcatheter edge‐to‐edge repair

Our regression analysis revealed that history of atrial fibrillation and arterial hypertension were inversely associated with the reduction of pulmonary artery pressures following M‐TEER. Patients with atrial fibrillation consistently exhibited poorer RV function and larger left atrial volumes at baseline, suggesting more advanced cardiac damage that developed in a non‐linear fashion independent of MR severity. Interestingly, while M‐TEER reduced left atrial volumes both in patients with and without atrial fibrillation, only those without atrial fibrillation experienced a significant reduction in sPAP levels. This suggests that the presence of atrial fibrillation may limit the potential for haemodynamic improvement, possibly due to chronic atrial dilatation and remodelling that are both less likely to be reversible. This finding aligns with an unsupervised machine learning study on the phenotyping of patients with severe MR, which showed that patients with atrial fibrillation, biatrial dilatation and development of severe tricuspid regurgitation had the worst prognosis after M‐TEER.^8^ Furthermore, evidence from the PARTNER trials on patients with severe aortic stenosis undergoing aortic valve replacement indicates those with arterial hypertension are less likely to recover from extra‐aortic valve cardiac damage – possibly because arterial hypertension has led to left ventricular remodelling and diastolic dysfunction which persisted even after aortic valve replacement.^29^ Taken together, our findings suggest that more aggressive management of atrial fibrillation and arterial hypertension is needed in patients with RV dysfunction to fully maximize the benefits of M‐TEER. Importantly, this applies to both primary and secondary MR, as we found no differences in the potential for sPAP reduction or RV functional recovery across aetiology subtypes.

### Limitations

Being a retrospective, observational, non‐randomized register study with inherent weaknesses, two major limitations of our analysis merit consideration. First, follow‐up echocardiography at 3 months after M‐TEER was available for only 55.8% of patients. The reasons for missing follow‐ups are speculative but could include logistical challenges, such as patients being referred from rural areas to the participating heart centres in Munich and Bad Oeynhausen, requiring long travel distances. This is particularly problematic for elderly, immobile patients (the median age of the study population was 79.9 years [IQR 74.5–83.5 years] at baseline). Additionally, some patients may have died between the procedure and follow‐up; however, the 1‐year survival rate of 86.8% (*Figure* *1*) suggests that this accounts for only a small proportion of cases. Importantly, it is unlikely that a selection bias was introduced by including only those patients who did not recover after M‐TEER and thus returned for follow‐up. On the contrary, survival analyses indicate that those who did not attend follow‐up had significantly worse outcomes, possibly due to ongoing right heart failure. It remains elusive whether including these patients, characterized by an elevated hazard ratio for 1‐year mortality of 5.04 (95% CI 3.23–7.86) (online supplementary *Figure Appendix* *S1*), would have demonstrated not only irreversible right heart dysfunction but also further deterioration. Second, although all echocardiographic studies were locally reviewed by experienced sonographers (VF and TT), no core laboratory was involved, which may have introduced interobserver variability in the measurement of cardiac structure and function. Additionally, blinding was not possible, as the presence of implanted devices at the mitral valve was clearly visible to the sonographer, potentially introducing bias in the assessment of RV recovery after M‐TEER. This underscores the importance of incorporating deep learning models for evaluating RV function, offering a reliable and unbiased assessment method. However, this study does not advocate replacing clinicians with artificial intelligence. The expertise of a cardiologist who integrates image acquisition and interpretation with clinical understanding remains irreplaceable.

Given the retrospective design of this study, the results should be interpreted as hypothesis‐generating rather than definitive. While our findings provide important insights into RV function following MV TEER, a prospective, randomized trial would be necessary to validate these results under controlled conditions, ensuring their clinical applicability and impact on guidelines.

## Conclusion

This study highlights that while M‐TEER improves left‐sided haemodynamics, such as reducing left atrial volume and pulmonary artery pressures, it does not lead to significant recovery of RV function. By employing a deep learning model to assess echocardiographic studies at baseline and follow‐up, we provided an objective, unbiased evaluation of RV function, adding robustness to our findings. The absence of RV functional recovery suggests that RV dysfunction may become irreversible in many patients, emphasizing the importance of early intervention, as waiting too long may limit the benefits of M‐TEER. Furthermore, comorbidities such as atrial fibrillation and arterial hypertension require more aggressive treatment to help patients fully realize the benefits of M‐TEER.

## Supporting information


**Appendix S1.** Supporting Information.
