# Supplementary material for: Employment of artificial intelligence for an unbiased evaluation regarding the recovery of right ventricular function after mitral valve transcatheter edge‐to‐edge repair
Source: Eur J Heart Fail. 2025 Jun 9;27(11):2452–64. doi: 10.1002/ejhf.3705 (PMC12765041; doi:10.1002/ejhf.3705)

Supplemental Material

Supplementary Table 1: Number of implanted devices per patient.

Number of clips per patient	No. (%)
**1 clip implanted**	567 (66.6%)
**2 clips implanted**	260 (30.6%)
**3 clips implanted**	24 (2.8%)

Supplementary Table 2: Implanted devices.

Device	No. (%)
**1st generation MitraClip (*Classic*)**	133 (15.6%)
**2nd generation MitraClip (*NT*)**	87 (10.2%)
**3rd generation MitraClip (*NTR, XTR*)**	128 (15.0%)
**4th generation MitraClip(*NT, NTW, XT, XTWXTR*)**	218 (25.6%)
**1st generation PASCAL**	168 (19.7%)
**PASCAL Ace**	117 (13.7%)

Supplementary Table 3: Comparison of baseline echocardiographic characteristics per etiology of mitral regurgitation.

	Etiology of mitral regurgitation			
	Primary (*n* = 299 patients)	Secondary (*n* = 457 patients)	*Mixed (n = 95 patients)*	*p*-value
**LVEF, %**	55 (50-60)	42 (30-53)	52 (42-60)	$<2.2x10^{-16}$
**LVEDD, mm**	54 (49-59)	59 (52-65)	51 (46-57)	$1.3x10^{-13}$
**LVESD, mm**	36 (31-42)	45 (37-55)	38 (32-44)	$<2.2x10^{-16}$
**LVEDV, mL**	118 (83-160)	162 (108-211)	126 (79-171)	$3.5x10^{-7}$
**LVESV, mL**	52 (34-77)	96 (54-150)	67 (35-102)	$1.6x10^{-14}$
**MV EROA, cm²**	0.44 (0.30-0.70)	0.27 (0.20-0.34)	0.30 (0.20-0.40)	$<2.2x10^{-16}$
**MR vena contract width, cm**	1.0 (0.8-1.2)	0.6 (0.5-0.8)	0.6 (0.6-0.7)	$1.9x10^{-13}$
**MV regurgitation volume, mL**	69 (51-88)	41 (32-53)	50 (37-63)	$<2.2x10^{-16}$
**LA volume, mL**	121 (93-165)	123 (91-169)	126 (98-171)	0.811
**sPAP, mmHg**	45 (35-58)	46 (35-58)	46 (37-56)	0.921
**Right midventricular diameter, mm**	29 (26-34)	32 (27-36)	30 (25-34)	0.002
**TAPSE, mm**	19 (15-22)	17 (13-20)	16 (13-19)	$3.3x10^{-7}$
**Predicted RVEF, %**	45.1 (41.2-49.6)	42.0 (37.6-45.7)	43.0 (39.9-46.2)	$6.2x10^{-10}$
**RA area, cm²**	24 (18-29)	26 (20-33)	25 (20-32)	0.004
**TAPSE/sPAP ratio, mm/mmHg**	0.404 (0.279-0.574)	0.355 (0.274-0.497)	0.360 (0.256-0.470)	0.025
**MR II and II+/IV°, No. (%)**	8 (2.7%)	21 (4.6%)	10 (10.5%)	0.006
**MR III and III+/IV°, No. (%)**	127 (42.5%)	307 (67.2%)	58 (61.1%)	$1.2x10^{-10}$
**MR IV/IV°, No. (%)**	164 (54.8%)	129 (28.2%)	27 (28.4%)	$2.1x10^{-13}$
**TR ≥III/IV°, No. (%)**	42 (14.0%)	124 (27.1%)	25 (26.3%)	$8.8x10^{-5}$

Categorical data are presented as numbers and frequencies (%), while continuous data are expressed as median and interquartile range.

LA volume: left atrial volume; LVEDD: left ventricular end-diastolic diameter; LVEDV: left ventricular end-diastolic volume; LVEF: left ventricular ejection fraction; LVESD: left ventricular end-systolic diameter; LVESV: left ventricular end-systolic volume; MR: mitral regurgitation; MV EROA: mitral valve effective regurgitant

orifice area; MV regurgitation volume: mitral valve regurgitation volume; RA area: right atrial area; RVEF: right ventricular ejection fraction; sPAP: systolic pulmonary artery pressure; TAPSE: tricuspid annular plane systolic excursion; TR: tricuspid regurgitation.

Supplementary Figure 1: Survival after M-TEER in patients with and without follow-up echocardiography.

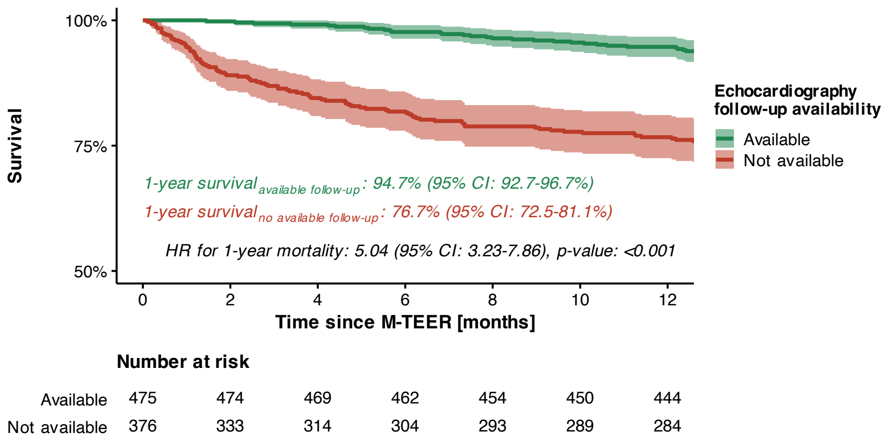

Supplementary Figure 2: Paired box plots comparing structural and functional hemodynamic changes following M-TEER stratified by etiology of mitral regurgitation.

LA volume: left atrial volume; LVEF: left ventricular ejection fraction; M-TEER: mitral valve transcatheter edge-to-edge repair; RVEF: right ventricular ejection fraction; sPAP: systolic pulmonary artery pressure; TAPSE: tricuspid annular plane systolic excursion.

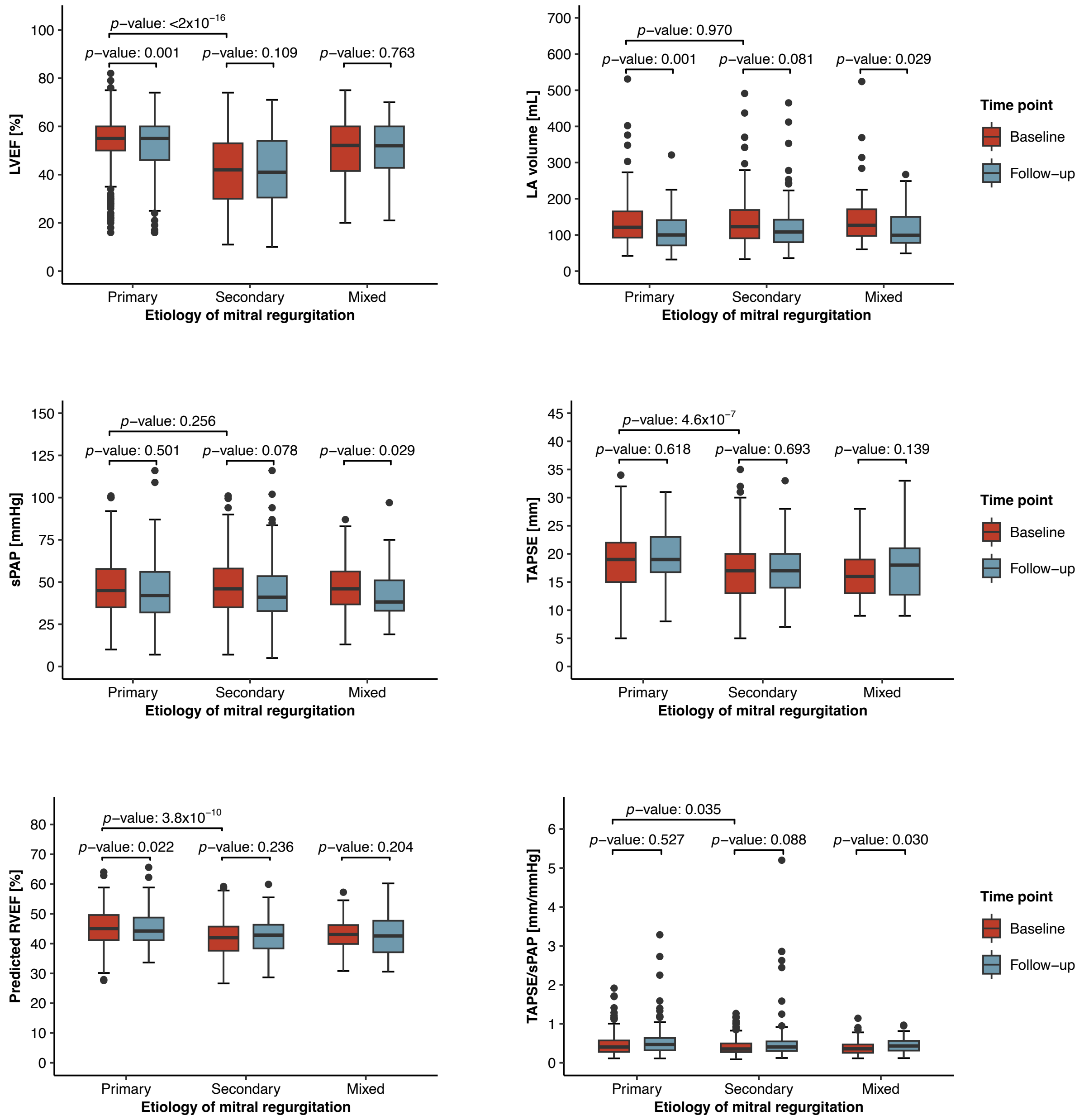

Supplementary Figure 3: Right ventricular function in relation to pulmonary hypertension.

A) Correlation plots illustrating the association between right ventricular function reduction and pulmonary hypertension, expressed as systolic pulmonary artery pressure. The blue line indicates the linear regression line, whereas the gray area denotes the 95% confidence interval.

B) Paired box plots comparing right ventricular function (TAPSE and deep learning-predicted RVEF) at baseline and follow-up after mitral valve transcatheter edge-to-edge repair, stratified by pulmonary hypertension severity.

LVEF: left ventricular ejection fraction; RVEF: right ventricular ejection fraction; sPAP: systolic pulmonary artery pressure; TAPSE: tricuspid annular plane systolic excursion.

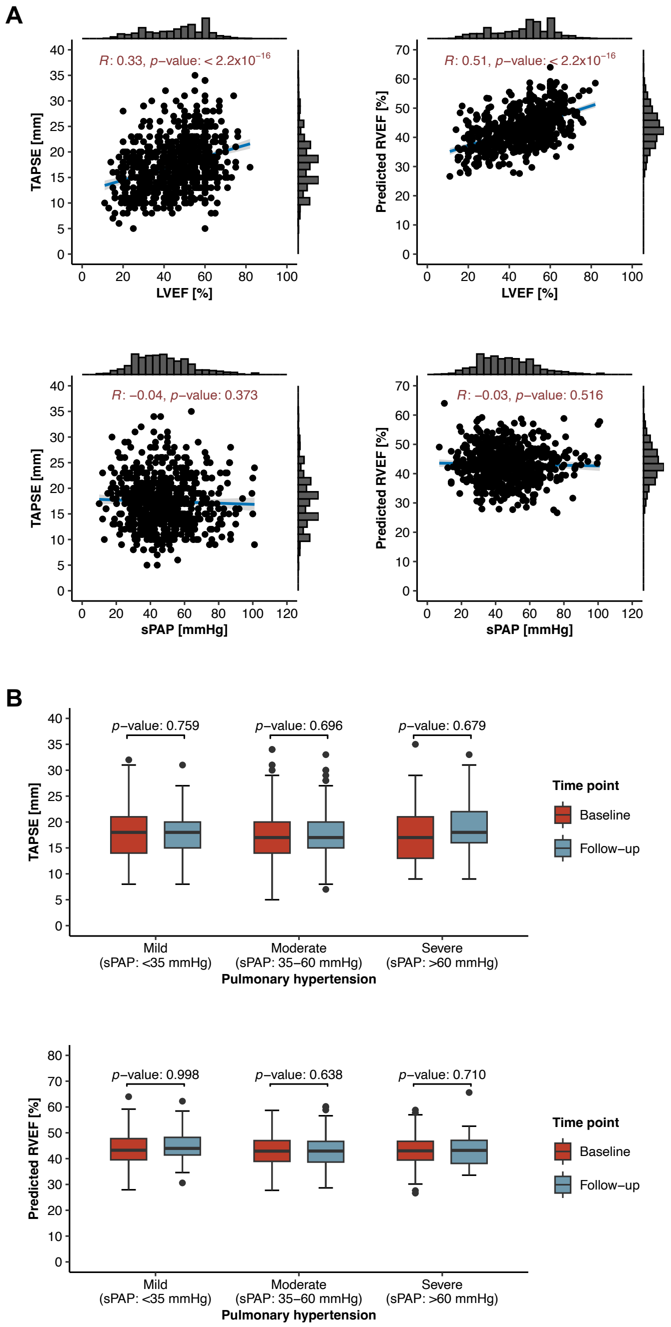

Supplement: Supplementary file 1 — Appendix S1. Supporting Information. [file EJHF-27-2452-s001.pdf]
